# Characteristics, clinical care, and outcomes of sepsis among patients boarding in the emergency department

**DOI:** 10.1002/jhm.13536

**Published:** 2024-10-27

**Authors:** Jessica A. Blank, Jessie E. King, Julieann F. Grant, Shuo Tian, Sachita Shrestha, Peter England, David Paje, Stephanie P. Taylor

**Affiliations:** ^1^ Department of Internal Medicine University of Michigan Ann Arbor Michigan USA; ^2^ Department of Internal Medicine, Division of Hospital Medicine University of Michigan Ann Arbor Michigan USA; ^3^ Department of Emergency Medicine University of Michigan Ann Arbor Michigan USA; ^4^ Institute for Health Policy and Innovation University of Michigan Ann Arbor Michigan USA

## Abstract

**Background:**

Patients who first meet clinical criteria for sepsis while boarding in the emergency department (ED) may not receive optimal sepsis care.

**Objective:**

Assess the association between ED boarding status and sepsis quality of care and outcomes.

**Methods:**

We conducted a retrospective cohort study of adult patients admitted to a large academic hospital from July 2021 to October 2023 who had clinical features consistent with sepsis present while physically in the ED. We compared outcomes for patients who experienced time zero (T‐0; the time clinical features of sepsis were first present) while boarding in the ED (physically in the ED but admitted to a different service) to those experiencing T‐0 while still under the care of the ED provider team. We used logistic regression to estimate the association between ED boarding status at T‐0 and compliance with the US Centers for Medicare & Medicaid Services (CMS) Severe Sepsis and Septic Shock: Management Bundle (SEP‐1) core measure, individual bundle element compliance, and hospital mortality adjusting for prespecified covariates. In a subgroup analysis among patients who had not already received antibiotics before T‐0, we conducted a Cox proportional hazards model to estimate the association between boarding status on time‐to‐antibiotics.

**Results:**

Among 4795 patients meeting a clinical definition of sepsis in the ED, 422 (8.8%) experienced T‐0 as ED boarders. These patients were similar in age, sex, and comorbidities compared with patients experiencing T‐0 while still under ED care. Fewer patients with T‐0 as an ED boarder received SEP‐1 compliant care (25% vs. 38%, *p* < .001), including a lower proportion of fluid resuscitation (15% vs. 26%, *p* = .004) and lactate assessment (62% vs. 94%, *p* < .001). Overall, more patients in the ED boarder group received antibiotics within 3 hours, but one‐third of patients had already received antibiotics prior to T‐0. Among patients who had not already received antibiotics prior to T‐0, experiencing T‐0 as an ED boarder was associated with a decreased likelihood of receiving antibiotics (hazard ratio [HR]: 0.67 [95% confidence interval [CI], 0.54–0.84]) and longer time to antibiotics from T‐0 (142 min vs. 100 min, *p* = .007).

**Conclusions:**

Sepsis patients experiencing T‐0 as a boarder in the ED have a lower likelihood of receiving SEP‐1 compliant care compared to patients who experience T‐0 while still under ED care.

## INTRODUCTION

Boarding in the emergency department (ED), defined as admitted patients remaining physically in the ED under non‐ED clinician care,^1^ has been associated with increased hospital mortality,^2^, ^3^ increased length of stay,^2^ and delays in appropriate treatment across multiple conditions.^4^ ED boarding contributes to ED crowding, which has been shown to impact both individual patient outcomes and health systems as a whole.^5^ As sepsis presents with heterogenous signs and symptoms, and early identification can be challenging, patients developing sepsis while boarding in the ED may experience delays in the implementation of critical life‐saving care. Evidence suggests that ED crowding, measured by the proportion of occupied beds, decreases the likelihood of adherence to the Surviving Sepsis Campaign bundle^6^ but the specific association between ED boarding on sepsis quality outcomes at the patient level has not been studied. We hypothesize that patients who meet “time zero” (T‐0), the time when sepsis is clinically identifiable, while boarding in the ED are less likely to receive care that is adherent to sepsis quality measures compared to patients who experience T‐0 while under ED care. We specifically chose this comparison group because, while ED boarding itself is difficult to modify, identification of care gaps for boarding patients provides a clear target for quality improvement efforts.

## METHODS

### Study setting and population

We identified adult (≥18 years old) patients with community‐acquired sepsis using Centers for Medicare & Medicaid Services (CMS) definitions for sepsis and followed CMS specifications to define T‐0, that is, the first point at which documented infection, organ dysfunction, and two systemic inflammatory response syndrome (SIRS) criteria occur within 6 h of each other.^6^, ^7^ We retrospectively extracted cohort data from July 2021 to October 2023 from the Michigan Medicine electronic data warehouse, including patient demographics, clinical characteristics, and treatment factors. We obtained data indicating patients' physical location and the service responsible for the patient at T‐0.

**Figure 1:**
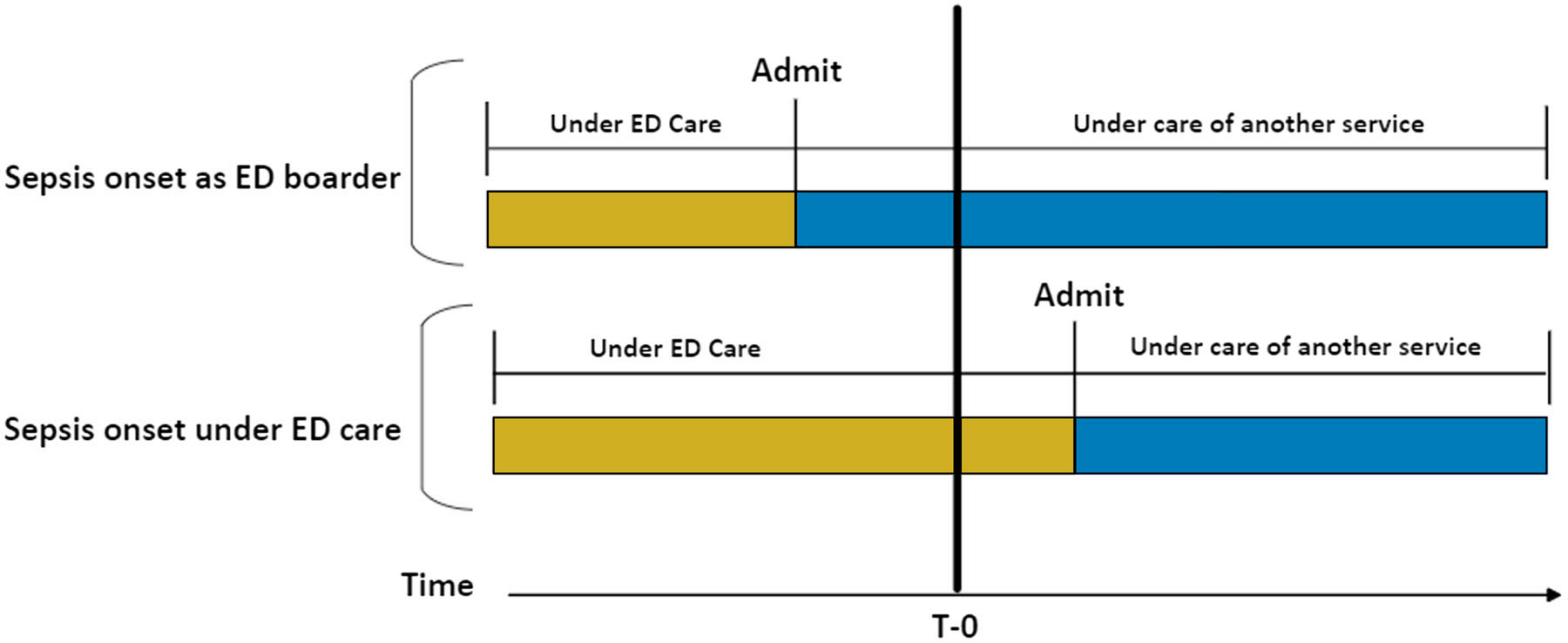
Schematic illustrating assignment of exposure and comparison groups based on the care delivery team at the onset of clinical features of sepsis.

Figure 1 shows a visual depiction of how exposure and comparison categories were defined. The primary exposure was whether a patient experienced T‐0 while boarding in the ED. We considered this exposure to be present when T‐0 occurred during a time that a patient's physical location was in the ED and the treatment service was a service other than Emergency Medicine. The comparison group was patients who experienced T‐0 while still under the treatment of Emergency Medicine. Since ED boarders may receive antibiotics before T‐0 when infection is suspected but sepsis criteria are not yet met, we conducted a subgroup analysis among patients who received antibiotics after T‐0. The study was approved by the University of Michigan Institutional Review Board (HUM00242849).

### Study outcomes

The primary outcome was SEP‐1 bundle compliance (antibiotic administration, lactate measurement, and blood culture collection within 3 h, and 30 cc/kg of intravenous fluid administration if hypotension or lactic acidosis is present). As per CMS specifications, antibiotics administered up to 24 h before T‐0 count toward the SEP‐1 antibiotic component. Secondary outcomes were individual bundle element compliance and hospital mortality. In a subgroup analysis of patients who received antibiotics after T‐0, we evaluated time‐to‐antibiotics from T‐0 as another outcome measure that could be a target for quality improvement.

### Data analysis

Descriptive statistics were summarized as median (interquartile range) for continuous variables, and count (percentage) for categorical variables. We used the Wilcoxon rank sum test, Fisher's exact test, and *χ* ^2^ test to compare differences between patients who were under ED care versus ED boarders, as well as the subset of patients who received antibiotics after T‐0. We used multivariable logistic regression to estimate the association between ED boarding status and SEP‐1 compliance, adjusting for a priori identified covariates of age, BMI, presence of septic shock (defined by CMS criteria: hypotension requiring vasopressors or a lactate ≥ 4 mmol/L within 6 h of T‐0), and Charlson comorbidity index.

For the subgroup analysis among patients who received antibiotics after T‐0, we examined time‐to‐antibiotics at 12 h follow‐up using a Cox proportional hazards model. We chose the 12 h follow‐up because although SEP‐1 uses a 3 h target for antibiotics, some data suggest that mortality benefits are seen at 6 h or longer.^8^, ^9^ We used a logistic regression model to estimate the association between ED boarding status and antibiotic compliance. All analyses were conducted using RStudio software version 2023.06.2.

## RESULTS

Of 4795 adult patients with sepsis onset in the ED, 422 (8.8%) experienced T‐0 while boarding in the ED, and 4373 (91.2%) experienced T‐0 while under ED care. Characteristics and outcomes for patients meeting T‐0 as ED boarders versus still under the care of the ED are described in Table 1. Groups were similar in age, gender, and comorbidities, but a lower proportion of ED boarders had a diagnosis of septic shock (21.3% vs. 33.1%, *p* < .001).

**Table 1:** Characteristics and outcomes of patients experiencing time zero while under ED care versus ED boarding.

Characteristic	Overall (*N* = 4795)	ED care (*N* = 4373)	ED boarder (*N* = 422)	*p* Value
Age, median (IQR)	64 (51, 74)	64 (52, 74)	64 (50, 74)	.73
Gender (female), *n* (%)	2063 (43.0%)	1880 (43.0%)	183 (43.4%)	.88
Race, *n* (%)				
White	3755 (78.3%)	3436 (78.6%)	319 (75.6%)	.16
Black	675 (14.1%)	607 (13.9%)	68 (16.1%)	.21
Other/unknown	365 (7.6%)	330 (7.5%)	35 (8.3%)	.58
BMI	26.7 (22.7, 32.3)	26.8 (22.7, 32.3)	26.5 (22.9, 31.1)	.38
Charlson Index, median (IQR)	9 (5, 15)	9 (5, 15)	9 (5, 15)	.83
CKD diagnosis, *n* (%)	1694 (35.3%)	1532 (35.0%)	162 (38.4%)	.17
HF diagnosis, *n* (%)	1438 (30.0%)	1311 (30.0%)	127 (30.1%)	.96
Septic shock, *n* (%)	1537 (32.1%)	1447 (33.1%)	90 (21.3%)	< .001
SEP‐1 compliance, *n* (%)	1760 (36.7%)	1654 (37.8%)	106 (25.1%)	< .001
Fluid resuscitation in 3 h if hypotensive or elevated lactate, *n* (%)	495 (24.9%)	474 (25.7%)	21 (14.9%)	.004
Lactate collected in 3 h, *n* (%)	4373 (91.2%)	4113 (94.1%)	260 (61.6%)	<.001
Antibiotics in 3 h, *n* (%)	3213 (67.0%)	2907 (66.5%)	306 (72.5%)	.01
Blood culture in 3 h, *n* (%)	3723 (77.6%)	3391 (77.5%)	332 (78.7%)	.60
Hospital mortality, *n* (%)	626 (13.1%)	574 (13.1%)	52 (12.3%)	.64
Hospital LOS (day), median (IQR)	8 (5, 15)	8 (5, 15)	9 (6, 16)	.04
ED LOS (hour), median (IQR)	18 (10, 28)	17 (10, 27)	26 (14, 33)	<.001

Abbreviations: CKD, chronic kidney disease; ED, emergency department; HF, heart failure; IQR, interquartile range; LOS, length of stay, T‐0, time zero.

Compared with patients with T‐0 while still under ED care, a lower proportion of patients with T‐0 while boarding received the full SEP‐1 bundle (25.1% vs. 37.8%, *p* < .001), including lower proportions of fluid resuscitation (14.9% vs. 25.7%, *p* = .004) and lactate measurement (61.6% vs. 94.1%, *p* < .001). The proportion of patients obtaining blood cultures was similar (78.7% vs. 77.5%, *p* = .60). A greater proportion of patients with T‐0 while boarding received antibiotics within 3 h (72.5% vs. 66.5%, *p* = .01).

Table 2 shows the association of ED boarding on SEP‐1 compliance and mortality adjusting for age, BMI, Charlson comorbidity index, and presence of septic shock. Experiencing T‐0 as an ED boarder versus still under the care of the ED (control group) was associated with a decreased likelihood of SEP‐1 compliance (odds ratio [OR]: 0.46 [95% confidence interval (CI), 0.36–0.58]), with no difference in hospital mortality (OR: 0.95 [95% CI, 0.66–1.34]).

**Table 2:** Effects of ED status on SEP‐1 compliance and hospital mortality.

Outcome (ED boarder vs. ED care)	OR	95% CI	*p* Value
SEP‐1 compliance	0.46	0.36, 0.58	< .001
Hospital mortality	0.95	0.66, 1.34	.79

*Note*: Adjusted for age, BMI, Charlson comorbidity index, and presence of septic shock.

Abbreviations: CI, confidence interval; ED, emergency department; OR, odds ratio.

### Subgroup analysis

One‐third of patients (35.3%) received antibiotics before T‐0 and could not contribute to an analysis of time‐to‐antibiotics. We identified a subgroup of patients who received antibiotics after T‐0 to examine the association between ED boarding status on recognition and response to sepsis onset in patients for whom infection was not already apparent prior to the emergence of clinical criteria for sepsis. Characteristics and outcomes of the subgroup analysis of patients who received antibiotics after T‐0 as ED boarders versus still under the care of ED clinicians are described in Table 3. The groups had no statistically significant difference in age, gender, comorbidities, or presence of septic shock.

**Table 3:** Characteristics and outcomes in the subgroup of patients who received antibiotics after time zero.

**Characteristic**	**Overall** (*N* = 3100)	**ED care** (*N* = 3023)	**ED boarder** (*N* = 77)	***p* Value**
Age, median (IQR)	64 (52.0, 74.0)	64 (52.0, 74.0)	63 (50.0, 73.0)	.33
Gender (female), *n* (%)	1321 (42.6%)	1288 (42.6%)	33 (42.9%)	.96
Race, *n* (%)				
White	2453 (79.1%)	2397 (79.3%)	56 (72.7%)	.16
Black	433 (14.0%)	417 (13.8%)	16 (20.8%)	.08
Other/unknown	214 (6.9%)	209 (6.9%)	5 (6.5%)	.89
BMI	26.8 (22.6, 32.4)	26.8 (22.6, 32.4)	25.0 (22.5, 29.6)	.09
Charlson Index, median (IQR)	9 (5.0, 15.0)	9 (5.0, 15.0)	9 (5.0, 14.0)	.90
CKD diagnosis, *n* (%)	1065 (34.4%)	1039 (34.4%)	26 (33.8%)	.91
HF diagnosis, *n* (%)	919 (29.6%)	892 (29.5%)	27 (35.1%)	.29
Septic shock, *n* (%)	1066 (34.4%)	1044 (34.5%)	22 (28.6%)	.30
SEP‐1 compliance, *n* (%)	1220 (39.4%)	1203 (39.8%)	17 (22.1%)	.002
Fluid resuscitation in 3 h if hypotensive or elevated lactate, *n*(%)	332 (24.8%)	331 (25.3%)	1 (3.2%)	.005
Lactate collected in 3 h, *n* (%)	2938 (94.8%)	2877 (95.2%)	61 (79.2%)	<.001
Antibiotics in 3 h, *n* (%)	2171 (70.0%)	2127 (70.4%)	44 (57.1%)	.01
Blood culture in 3 h, *n* (%)	2514 (81.1%)	2460 (81.4%)	54 (70.1%)	.01
T‐0 to antibiotics (Min), median (IQR)	101 (46, 214)	100 (45, 212)	142 (64, 325)	.007
Hospital mortality, *n* (%)	404 (13.0%)	393 (13.0%)	11 (14.3%)	.74
Hospital LOS (day), median (IQR)	8 (5, 14)	8 (5, 14)	9 (6, 14)	.09
ED LOS (hour), median (IQR)	17 (10, 27)	17 (10, 27)	20 (11, 29)	.52

Abbreviations: BMI, body mass index; CKD, chronic kidney disease; ED, emergency department; HF, heart failure; IQR, interquartile range; LOS, length of stay; T‐0, time zero.

**Figure 2:**
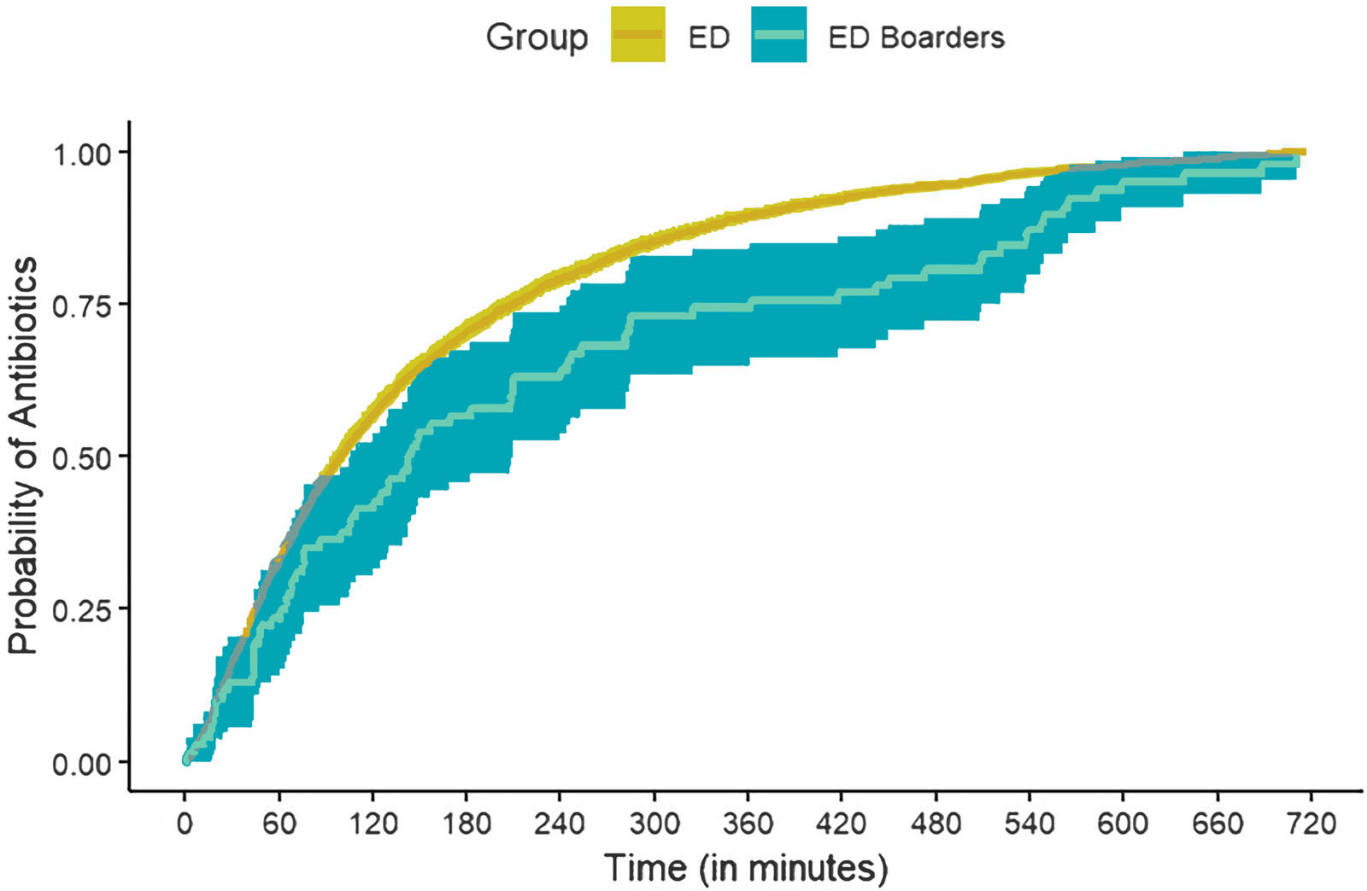
Cumulative curves of antibiotics given within 12 h of time zero for patients who received antibiotics after time zero. The curves were plotted using the Cox proportional hazards model showing time‐to‐antibiotics for patients experiencing time zero as ED boarders and patients experiencing time zero under ED care, adjusting for age, BMI, Charlson comorbidity index, and presence of septic shock. BMI, body mass index; ED, emergency department.

Compared with patients still under ED care, a lower proportion of ED boarders received SEP‐1 compliant care (22.1% vs. 39.8%, *p* = .002) and individual bundle elements including fluid resuscitation (3.2% vs. 25.3%, *p* = .005), lactate measurement (79.2% vs. 95.2%, *p* < .001), blood cultures (70.1% vs. 81.4%, *p* = .01), and antibiotics within 3 h (57.1% vs. 70.4%, *p* = .01). The median time from T‐0 to receiving antibiotics was longer for patients in the ED boarding group than for those with T‐0 while under ED care (142 min vs. 100 min, *p* = .007). In the multivariable Cox proportional hazards model, patients with T‐0 as an ED boarder had a decreased cumulative probability of receiving antibiotics within 12 h of T‐0 (adjusted hazard ratio 0.67 [95% CI, 0.54–0.84]) compared with patients meeting T‐0 while under ED care (Figure 2).

## DISCUSSION

We found that among adult patients with community‐acquired sepsis, those meeting T‐0 while boarding in the ED received SEP‐1 compliant care less frequently, consistent with data showing lower quality of care among boarders experiencing other conditions.^10^, ^11^ Examining individual SEP‐1 bundle elements, ED boarders were less likely to receive lactate measurement and bundle‐compliant fluid resuscitation. Although ED boarders overall were more likely to receive antibiotics within 3 h, this was driven by ED boarders frequently receiving antibiotics before T‐0, before clinically diagnosed sepsis. In a subgroup of patients who had not already received antibiotics before T‐0, ED boarders had longer time‐to‐antibiotics and were 43% less likely to receive antibiotics within 3 h.

The identified deficits in sepsis care for ED boarders have a significant impact due to the prevalence of community‐acquired sepsis and the increasing rates of ED boarding—in our cohort nearly 10% of sepsis patients experienced T‐0 while boarding. As sepsis is a leading cause of hospitalization and hospital mortality, addressing sepsis quality gaps in the setting of ED boarding is critical, including early and accurate diagnosis of sepsis in the ED as well as appropriate processes for recognition and response to sepsis that is identified during boarding.

Hospitals can consider several approaches to improving this care gap for sepsis patients. Foremost, hospitals should continue broad efforts to increase inpatient capacity and decrease ED boarding. Other potential solutions might include the creation of localized medical teams for ED boarders that can provide closer monitoring and faster response times when patients meet the criteria for sepsis.^12^ Accurate early identification tools could provide prompt notification to the primary team when ED boarding patients meet sepsis criteria, but work is needed to improve the accuracy of such tools and understand important contextual factors for implementation success including both patient and clinician level factors.

Limitations of the study include that it was conducted at a single academic center. Settings with different ED boarding rates, practices, and patterns of sepsis care may have different results. Additionally, although we accounted for prespecified covariates of age, BMI, Charlson comorbidity index, and presence of septic shock, the results could be biased by unmeasured confounding variables. We reported mortality outcomes, but our study was not powered to detect downstream health outcomes such as mortality.

## CONCLUSIONS

Patients who meet T‐0 for community‐acquired sepsis while boarding in the ED have lower rates of SEP‐1 compliance. Understanding quality gaps in sepsis treatment for ED boarders is important to improve the quality of sepsis care.

## CONFLICTS OF INTEREST STATEMENT

S. P. T. received grant funding from AHRQ related to this work, grant funding from NINR and NHLBI, and consulting fee from Abionyx Biotech outside the scope of this work. The remaining authors declare no conflict of interest.

## ETHICS STATEMENT

The study was approved by the University of Michigan Institutional Review Board (HUM00242849).

## Data Availability

The data that support the findings of this study are available from the corresponding author upon reasonable request.
